# Prooxidant effect of uric acid on human leukocytic DNA: An in vitro and ex vivo study

**DOI:** 10.55730/1300-0152.2735

**Published:** 2025-01-02

**Authors:** Yim Tong Savio SZETO, Vincy Sze Wing LI, Yuen Lam PON

**Affiliations:** 1 School of Medical and Health Sciences, Tung Wah College, Homantin, Hong Kong; 2 Department of Pathology, Tseung Kwan O Hospital, Hong Kong; 3 Clinical Genetics Service Department, Hong Kong Children’s Hospital, Hong Kong

**Keywords:** Antioxidant, comet assay, DNA, prooxidant, urate

## Abstract

**Background/aim:**

Uric acid is a major contributor to the total antioxidant capacity of human plasma. However, this endogenous substance’s antioxidant and prooxidant properties have not yet been reported.

**Materials and methods:**

In this study, the comet assay was employed in vitro to determine the effect of uric acid on DNA damage in human lymphocytes and leukocytic DNA damage in hyperuricemia patients with and without renal failure.

**Results:**

DNA damage in lymphocytes occurred at uric acid concentrations of ≥600 μM. Adding catalase to the uric acid solution diminished the damaging effect, indicating that hydrogen peroxide mediated the prooxidant activity. Moreover, adding Fe^2+^ did not enhance the DNA damage, suggesting that the urate’s prooxidant activity is independent of the Fenton reaction. The unstable nature of uric acid at nearly neutral and acidic pH levels resulted in autooxidation and the generation of hydrogen peroxide. Maintaining the stability of uric acid in vivo may lead to the consumption of antioxidants in the body and affect the antioxidant status. Hyperuricemia patients with and without renal failure had higher levels of leukocytic DNA damage compared to healthy individuals. However, there was no significant difference in leukocytic DNA damage between hyperuricemia patients with and without renal failure, which showed that the damaging effect was not due to renal failure. A correlation study suggested that serum uric acid level had a stronger correlation with DNA damage than the severity of renal failure as indicated by serum creatinine or urea.

**Conclusion:**

Uric acid demonstrated prooxidant activity in both in vitro and in vivo studies, which was mediated by the production of hydrogen peroxide and independent of both the Fenton reaction and renal failure.

## 1. Introduction

Uric acid is generally regarded as a waste product but it is also an effective antioxidant in vitro (Davie et al., 1986; Álvarez-Lario and MacArrón-Vicente, 2011; Du et al., 2024). Uric acid has been suggested to replace ascorbic acid in primate evolution (Ames et al., 1981). It has also been proposed as a physiological antioxidant that can scavenge hydroxyl and superoxide radicals and inhibit lipid peroxidation (Stinefelt et al., 2005). It is a major contributor to serum total antioxidant capacity, and it is estimated that about 67% of the total antioxidant power in plasma is contributed by uric acid (Duplancic et al., 2011). However, a prooxidant role of uric acid has also been suggested. Recycling of the urate radical may consume the reducing power of ascorbic acid and lead to insufficient reducing power for effective recycling of oxidized α-tocopherol (Benzie and Strain, 1996). Evidence of prooxidant properties includes the formation of the free radical metabolite from uric acid (Maples and Mason, 1988). In addition, in the presence of Cu^2+^, uric acid has been shown to cause DNA strand scission (Shamsi et al., 1996). Generation of the reactive oxygen species and reactive nitrogen species by human fibroblast-like synoviocytes in the presence of monosodium urate has also been reported (Zamudio-Cuevas et al., 2016).

The primary diseases associated with uric acid are gout and uric acid nephrolithiasis (Harris et al., 1999). Hyperuricemia can result from overproduction of uric acid in the liver. Contributing factors include a purine-rich diet, alcoholism, and certain drugs, which can lead to increased uric acid production. Hyperuricemia can also be linked to hematological disorders such as myeloproliferative and lymphoproliferative disorders, as well as the use of cytotoxic drugs (Harris et al., 1999; de Oliveira and Burini, 2012). Renal failure is often associated with hyperuricemia due to decreased excretion by the kidneys. Over 60% of chronic kidney disease cases are accompanied by hyperuricemia (Richette and Bardin, 2010). Chronic kidney disease, a prevalent condition, affects approximately 14% of adults in the United States (Centers for Disease Control and Prevention, 2023). There is also evidence suggesting the presence of DNA damage in chronic kidney disease (Schupp et al., 2016), with renal tubular epithelial cells exhibiting higher levels of DNA damage in these conditions (Wang et al., 2023).

Uric acid is considered a double-edged sword (Li et al., 2022), as it behaves differently at normal and high plasma concentrations and regulates the aging process of endothelial cells. In vitro and ex vivo testing of human nuclear DNA was performed in this study to elucidate uric acid’s antioxidant and prooxidant activities in humans. The study aimed to investigate the in vitro effect of uric acid on human lymphocytes in terms of DNA damage or protective effects, if any. The contribution of oxidative stress in peripheral white blood cells in hyperuricemia patients with and without renal failure was also tested. DNA scores obtained by comet assay were used to determine leukocytic DNA levels.

## 2. Materials and methods

### 2.1. Chemicals and reagents

Chemicals and reagents of molecular biology grade were purchased. Type VII low-gelling-point agarose, standard agarose, phosphate-buffered saline (PBS) tablets, sodium chloride, disodium ethylenediaminetetraacetic acid dihydrate (EDTA), Tris(hydroxymethyl)aminomethane (Tris), hydrogen peroxide solution, ethidium bromide, Triton X-100, uric acid, and catalase were from Sigma-Aldrich (St. Louis, MO, USA). Disodium hydrogen phosphate, sodium dihydrogen phosphate, and sodium hydroxide were from Riedel-de Haen (Niedersachsen, Germany). Hydrochloric acid was from Merck (Darmstadt, Germany) and Giemsa stain was from BioGnost (Medjugorska, Croatia).

### 2.2. Participants

Healthy participants and participants with hyperuricemia were recruited in the clinic for the study. The health status of the participants was identified based on blood glucose, renal function, and liver function test results. Informed consent was obtained. Ethical approval of the study was granted by the Macao Society for the Study of Women’s Health.

### 2.3. In vitro study of effects of uric acid on lymphocytic DNA damage

#### 2.3.1. Preparation of lymphocytes

Lymphocytes from healthy participants (both male and female) were used as reagent cells. As previously described, lymphocytes were harvested and cryopreserved from fresh heparinized venous blood samples (Szeto and Benzie, 2002). Cells were kept at −80 °C and used within 6 weeks.

#### 2.3.2. Effect of uric acid on DNA damage

Fresh solutions of uric acid with and without catalase and an aged uric acid solution were prepared. The fresh uric acid solution in PBS (0.01 M, pH 7.4) was prepared with the assistance of sonication and was used within 1 h after preparation. The aged uric acid solution was kept in the dark at 4 °C for 1 week before use. Cells were washed in PBS, centrifuged for 4 min (1500 rpm, 4 °C) once in PBS, and incubated in 1 mL of uric acid solution at various concentrations (0–1000 μM) for 30 min at 37 °C. In separate experiments, a catalase suspension (3 μL, 230 IU) was added to 1 mL of uric acid solution at 0, 600, 800, and 1000 μM. Cells were washed once in cold PBS after incubation and a comet assay was performed following a previously described procedure (Szeto and Benzie, 2002). Slides were stained with ethidium bromide and image analysis proceeded without delay after the staining of each slide.

#### 2.3.3. Effect of low concentrations (200 and 400 μM) of uric acid on DNA damage

Since no detectable DNA damage was observed with the low-concentration uric acid treatment in the previous experiment, a modified enzyme-assisted comet assay was conducted to detect oxidized bases. To increase the sensitivity of the comet assay for measuring direct strand breaks as well as oxidized bases in DNA after incubation with a low concentration of uric acid, a step of incubating lysed cells with endonuclease III was included in the comet assay. After cells on glass slides were lysed with a lysis solution, the slides were transferred to a Coplin jar containing an enzyme buffer at 4 °C for 10 min. Subsequently, 50 μL of endonuclease III solution was added to each gel and the slides were covered with a cover slip. Control gels were incubated with the enzyme only. Slides were kept in a moist chamber at 37 °C for 45 min to induce DNA strand breaks on oxidized bases (Collins et al., 1996). The coverslip was removed at the end of incubation and slides were subjected to alkaline treatment as previously described (Szeto and Benzie, 2002).

#### 2.3.4. Effect of uric acid with Fe^2+^ on DNA damage

The demonstrated prooxidant effect was probably mediated by hydrogen peroxide. Therefore, Fe^2+^ was added to the uric acid solution to determine whether the Fenton reaction was involved in the prooxidant effect. A uric acid solution was prepared in 0.85% saline with the addition of a small amount of NaOH solution during sonication to assist in dissolving. The pH value of this solution (600 μM) was checked and did not exceed 7.5. The uric acid solution was used within 1 h after preparation. Cells were washed once in 0.85% saline (1500 rpm, 4 min, 4 °C) and incubated in 1 mL of uric acid and FeSO_4_ solution at various concentrations (final concentrations of uric acid: 0, 200, 400, 600 μM; FeSO_4_: 50 μM) for 30 min at 37 °C. After incubation, cells were washed once in cold PBS and then the comet assay was performed.

#### 2.3.5. Effect of uric acid on DNA damage at different pH levels

Since the stability of uric acid at near-neutral pH is lower than that at high pH (unpublished data), the product formed after the oxidation of uric acid might contribute to the prooxidant effect on DNA. Uric acid solutions were prepared at different pH levels to investigate whether the prooxidant effect was enhanced at low pH. Since uric acid being dissolved in PBS (pH 7.4) lowers the pH of the solution to 7.1, NaOH solution was added to 1000 μM uric acid solution to investigate the effect of uric acid at different pH levels. In this process, 0.1 M NaOH solution was used and the final pH of the uric acid solutions was 7.1 (no NaOH added), 7.2, 7.3, 7.4 and 7.8, while PBS (pH 7.4) was included as a control. Cells were washed once with cold PBS and then the comet assay was performed.

#### 2.3.6. Quality control of comet assay

Cryopreserved lymphocytes from three participants were treated with 0, 15, 30, and 60 μM hydrogen peroxide and used as control cells. Cells were treated with hydrogen peroxide in PBS for 5 min on ice and then subjected to the comet assay.

#### 2.3.7. Scoring of DNA damage

In the in vitro study, 50 cells were graded per gel. The tail DNA content (%) of each cell was measured using a computerized image analysis system (Komet 3.0, Kinetic Imaging, Liverpool, UK) under a fluorescence microscope (Optiphot-2, Nikon, Tokyo, Japan) fitted with a 580-nm emission filter. For the ex vivo study, visual scoring was performed under a light microscope. One hundred cells per gel were scored and classified on a scale of 0 to 4, with a score of 0 indicating intact DNA and a score of 4 indicating severely damaged DNA. The final comet score was the sum of the scores for 100 cells. Diagrams of this grading system are available in the literature (Chan et al., 2023).

### 2.4. Association of hyperuricemia and leukocytic DNA damage

The mean comet score obtained in the laboratory for healthy participants was approximately 130, with a standard deviation of 25. We aimed to detect a difference of 25 in the group of patients with hyperuricemia. With power of 80%, it was estimated that 16 participants would be required in each group. Fifty-five specimens were categorized into three groups: 20 from healthy individuals without any known diseases, 20 from patients with only hyperuricemia, and 15 from patients with both hyperuricemia and renal failure. Serum uric acid levels above 428 μM for men and 357 μM for women were considered indicative of hyperuricemia. Serum creatinine levels above 90 μM for both men and women were regarded as indicative of renal failure. Blood samples collected in EDTA tubes were stored at 4 °C for 1 to 2 days before being transferred to long-term storage at −20 °C until comet assay testing.

The whole-blood comet assay procedure without UV irradiation was adopted from a previous study (Chan et al., 2023). Two gels were prepared on one slide for each specimen. After electrophoresis, the slides were immersed in a staining jar with tap water for 5 min, which was subsequently changed 3 times to remove the electrophoresis solution. Slides were dried at 37 °C for 30 min and fixed in 70% ethanol for 10 min. Giemsa solution was filtered and diluted with phosphate buffer (pH 6.8) at a ratio of 1:1 before use. Fresh working Giemsa stain was added to the slides for 30 min followed by brief rinsing under tap water and destaining in phosphate buffer (pH 6.8) for 1–2 s. Slides were further rinsed with tap water and dried at 37 °C. Slides were scored as previously described (Chan et al., 2023).

### 2.5. Statistical analysis

Dunnett’s test was used to compare the DNA damage in cells treated with different concentrations of fresh or aged uric acid solutions at various pH levels. An unpaired t-test, suitable for identifying differences between the means of two groups, was employed to compare the DNA damage in cells treated with uric acid with and without endonuclease III and in cells treated with uric acid with and without Fe^2+^. The Kruskal–Wallis test with Dunn’s multiple comparison post hoc test was used to compare ages and comet scores among three groups: hyperuricemia with renal failure, hyperuricemia only, and healthy participants. Values of p < 0.05 were considered indicative of a significant difference in all statistical analyses. Linear regression was conducted to investigate the association between comet score and levels of uric acid, creatinine, and urea. Multivariate analysis was performed to check for any confounding effects among different analytes.

## 3. Results

**Figure 1:**
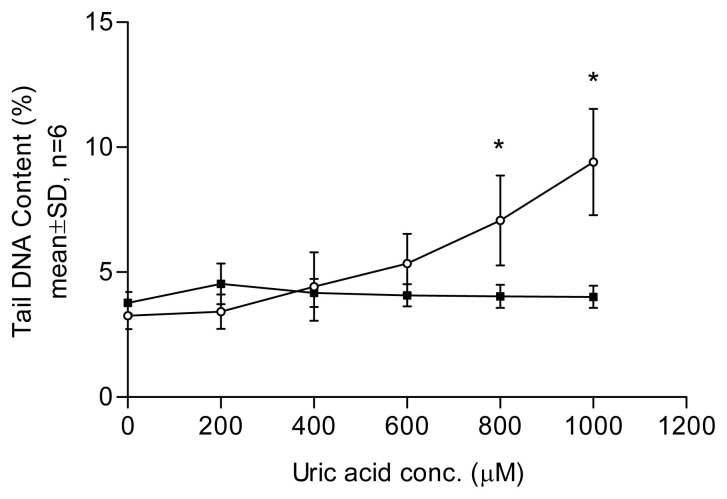
Effect of fresh and aged uric acid solution pretreatment on lymphocytes. Results were obtained using samples taken from three healthy participants and tested in two individual experiments. Each point represents the mean of tail %DNA content with ±1 SD error bars shown. DNA damage was positively related to concentration in fresh uric acid solution. A statistically significant difference was found at concentrations of ≥800 μM compared to 0 μM (Dunnett’s test, *: p < 0.05). No DNA damage was observed in cells incubated with aged uric acid solution at various concentrations (Dunnett’s test, p > 0.05). White circles represent fresh uric acid; black squares represent aged uric acid.

**Figure 2:**
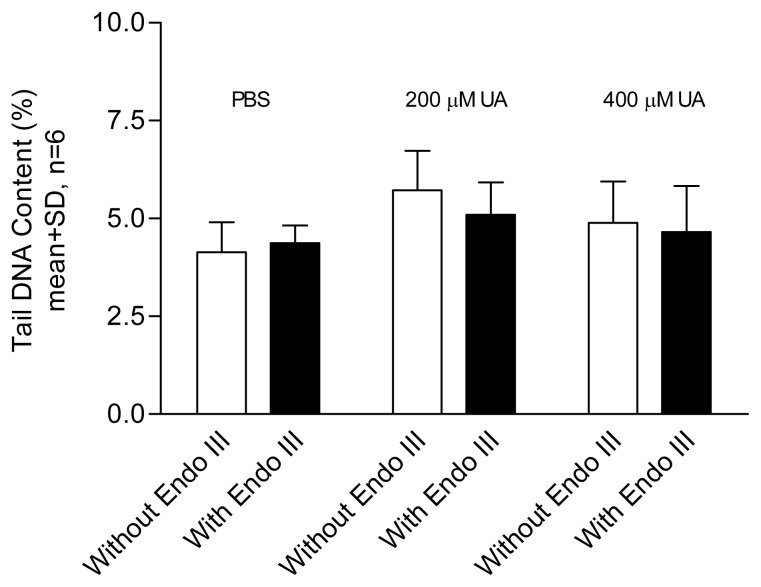
Effect of uric acid (UA) on lymphocytes with and without treatment by endonuclease III (Endo III). Results were obtained using samples taken from three healthy participants and tested in two individual experiments. Each bar represents the mean of tail %DNA content with +1 SD error bars shown. No statistically significant difference was found between cells treated with uric acid with or without the addition of treatment by endonuclease III (unpaired-t test, p > 0.05). White bars denote the lack of endonuclease III pretreatment; black bars denote the presence of endonuclease III pretreatment.

**Figure 3:**
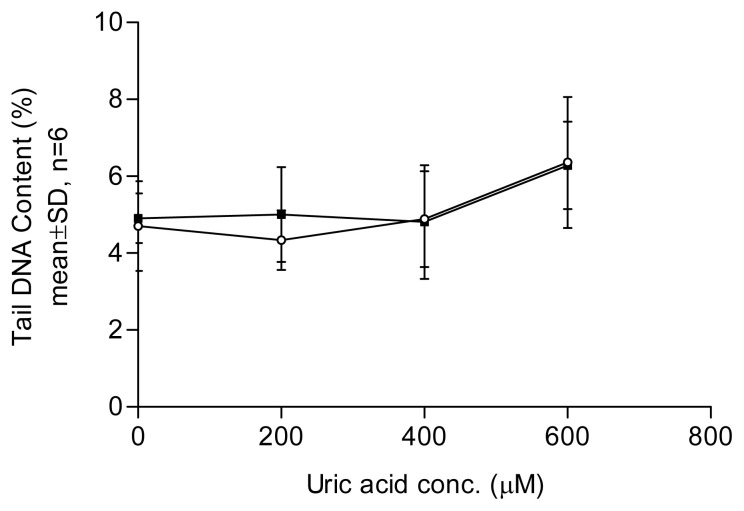
Effect of uric acid on lymphocytes with and without the presence of Fe^2+^. Results were obtained using samples taken from three healthy participants and tested in two individual experiments. Each point represents the mean of tail %DNA content with ±1 SD error bars shown. No statistically significant difference was found between cells treated with uric acid with and without adding Fe^2+^ (unpaired-t test, p > 0.05). White circles represent lack of Fe^2+^; black squares represent presence of 50 μM Fe^2+^ (final concentration).

**Figure 4:**
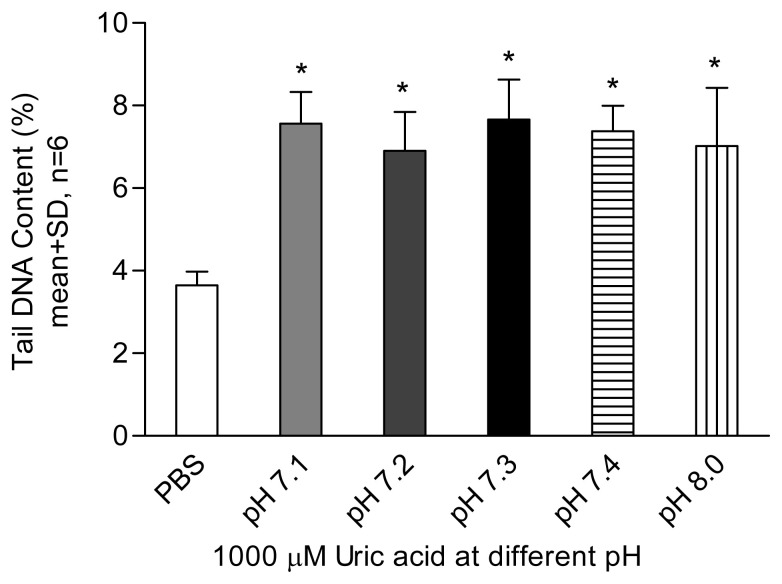
Effect of uric acid on lymphocytes at different pH levels. Results were obtained using samples taken from two healthy participants and tested in three individual experiments. Each bar represents the mean of tail %DNA content with +1 SD error bars shown. A statistically significant difference was found between cell pretreatment with uric acid at various pH levels compared to PBS at 7.4 (Dunnett’s test, *: p < 0.05).

**Figure 5:**
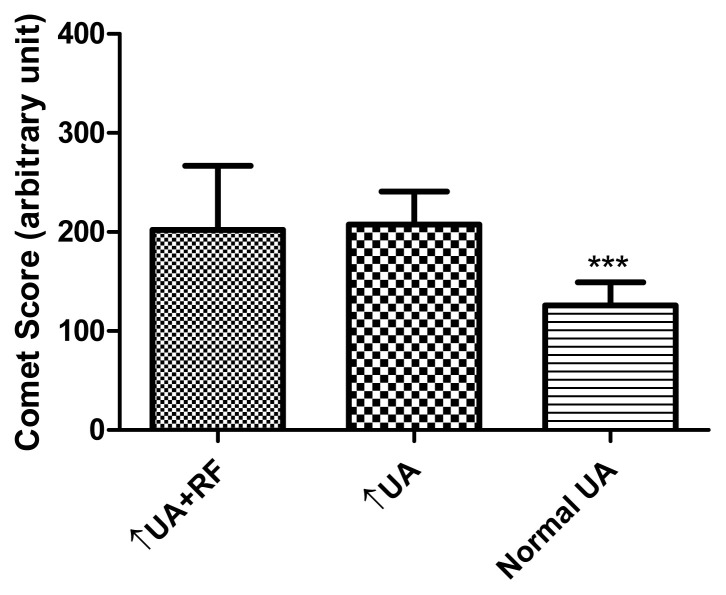
DNA damage according to comet scores for participants with hyperuricemia and renal failure (↑UA+RF; 202.0 ± 64.7, n = 15), participants with only hyperuricemia (↑UA; 207.0 ± 33.3, n = 20), and healthy participants (Normal UA; 125.0 ± 23.8, n = 20). ***: p < 0.05 (size effects from 1.7 to 2.9), with the values of healthy participants being statistically significantly lower than those of both hyperuricemia groups.

**Figure 6:**
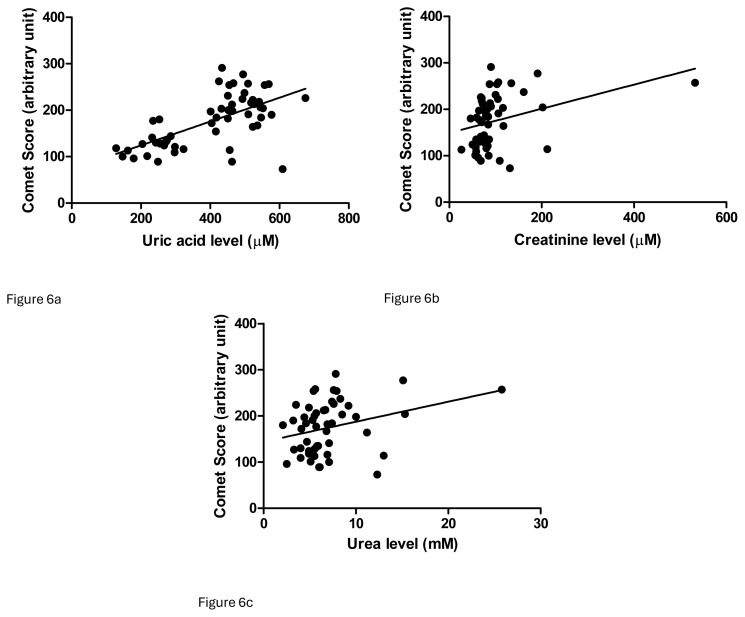
Linear regression studies demonstrated significant associations of DNA damage with **(a)** uric acid (r^2^ = 0.3939, p < 0.0001), **(b)** creatinine (r^2^ = 0.1107; p = 0.0159), and **(c)** urea levels (r^2^ = 0.0893; p = 0.0314).

In the comet assay, loops of DNA containing single- or double-stranded breaks are drawn out of the nuclei of lysed cells by an electric field, forming a comet-like tail. The amount of DNA in the tail correlates with the level of DNA damage incurred. Tail DNA content (Figures 1–4) and comet scores (Figures 5 and 6) represent the extent of DNA damage.

### 3.1. In vitro study of effects of uric acid on lymphocytic DNA

Uric acid at 600 μM began to show a DNA-damaging effect in lymphocytes, with a dose–response relationship observed (Figure 1). An aged uric acid solution containing no detectable uric acid had no adverse impact on DNA (Figure 1). The enzyme-assisted comet assay detected oxidized pyrimidines at uric acid treatment levels of 200 and 400 μM. Endonuclease III, used in the enzyme-assisted comet assay, did not show detectable oxidized pyrimidines at such low concentrations of uric acid treatment (Figure 2). The results suggest that the prooxidant effect was due to the presence of the uric acid itself rather than impurities in the solution. In the presence of catalase, no DNA damage was detected at any uric acid concentration tested, indicating that hydrogen peroxide played a role in the DNA-damaging effects. In the experiment investigating the effect of Fe^2+^, no significant difference was observed between the results obtained with and without Fe^2+^ addition (Figure 3). Uric acid at 1000 μM caused similar levels of DNA damage regardless of the solution’s pH. Cells incubated in PBS at pH 7.4 and pH 8.0 showed no significant difference in DNA damage (Figure 4).

### 3.2. Association of hyperuricemia and leukocytic DNA damage

The demographics and laboratory data of the participants are presented in Table 1. There was no statistically significant difference in age among the three groups (Kruskal–Wallis test, p = 0.0921, followed by Dunn’s multiple comparison test, p > 0.05). The results showed that hyperuricemia patients with renal failure had levels of leukocytic DNA damage similar to those of the hyperuricemia-only patients. Both groups demonstrated higher DNA damage compared to the control group (Dunn’s multiple comparison test, p < 0.05) (Figure 5).

Linear regression analyses were performed for uric acid, creatinine, and urea levels and comet scores. The coefficients of determination were as follows: comet score vs. uric acid = 0.3939, p < 0.0001; comet score vs. creatinine = 0.1107, p = 0.0159; comet score vs. urea = 0.0893, p = 0.0314 (Figure 6). However, in multivariate analysis, the positive associations between these parameters and comet scores were diminished.

## 4. Discussion

The results of this study indicated that the in vitro prooxidant effect of uric acid was mediated by hydrogen peroxide production since DNA damage was abolished by catalase. The addition of Fe^2+^ ions did not enhance the prooxidant effect of the uric acid. Previously, uric acid was reported to have the ability to bind iron ions (Davies et al., 1986). This intrinsic ability of uric acid may prevent the occurrence of the Fenton reaction despite the involvement of H_2_O_2_ in its prooxidant properties.

Uric acid is more stable in alkaline than near-neutral pH environments. At a concentration of 1000 μM, uric acid completely decomposed in PBS with a final pH of 7.1 at 4 °C in the dark for 48 h, while only 50% of the uric acid decomposed at pH 9.0 (unpublished data). The results showed that uric acid at pH levels of 7.1 to 8.0 exerted similar levels of DNA damage (Figure 4), indicating that uric acid stability cannot be maintained at physiological pH in a serum-free environment. To prevent prooxidant effects, antioxidants in the body may be consumed to protect uric acid from autooxidation, leading to additional oxidative stress.

The enzyme-assisted comet assay was used to detect low-level DNA damage. Endonuclease III can induce DNA strand breaks involving oxidized pyrimidines (Collins et al., 1997). Thus, DNA with direct strand breaks and oxidized bases can be detected using the comet assay. In this study, low concentrations of uric acid (400 μM or below) were not strong enough to induce direct DNA damage or a detectable amount of oxidized bases. The human serum level of uric acid is approximately 150–450 μM and DNA damage in the comet assay was observed at 600 μM (Figure 1), supporting the conclusion that high concentrations of uric acid may exert a more prooxidant than antioxidant effect (Cutler, 1991).

It should be noted that uric acid in vitro (i.e. in PBS) may not behave the same as it does in vivo since it has been reported that uric acid is stable in serum (Heins et al., 1995). However, maintaining uric acid stability in vivo may lead to the consumption of other antioxidants, resulting in the indirect depletion of antioxidant status. It has been suggested that the prooxidant effect contributed by uric acid is a more significant factor than the uric acid itself (Gherghina et al., 2022).

Inhibition of uric acid formation is likely preferable to enhanced uric acid excretion in treatment. Nevertheless, the role and action of uric acid as a prooxidant or antioxidant may depend on the specific situation and concentration, and further study of this interesting endogenous compound is required.

It has been suggested that patients with renal failure have higher levels of DNA damage in renal tubular epithelial cells as well as blood cells (Rangel-López et al., 2013; Wang et al., 2023). The present study demonstrated renal failure in hyperuricemia patients with higher levels of leukocytic DNA damage (Figure 5), but hyperuricemia patients with normal renal function presented with the same level of DNA damage. In other words, renal failure did not introduce extra oxidative stress for DNA. A regression study showed a stronger association between uric acid level and comet score than between creatinine or urea levels and comet score (Figure 6), but these significant correlations diminished in multivariate analysis. No particular parameters demonstrated a strong association with the comet score in multivariate analysis (Table 2). Uric acid can be a source of detrimental effects on organs (Copur et al., 2022). Humans are prone to hyperuricemia, which contributes more oxidative stress than renal failure, but more evidence is required. The relationships of blood uric acid with different oxidative stress markers are conflicting, as is the antioxidant status. It was suggested that serum uric acid and biological antioxidant potential were positively associated in a study group including over 5000 individuals (Ok et al., 2018). However, uric acid is always an antioxidant according to measurements of the reducing ability of Fe^3+^ to Fe^2+^ (Szeto and Benzie, 2002). Some other studies have concluded that uric acid is positively associated with oxidative stress in both humans and rat models (Sánchez-Lozada et al., 2012; Gormally et al., 2019).

The prooxidant effect of uric acid demonstrated in this in vitro study was observed within a serum-free environment. The possibility of the reduction of the prooxidant effect in vivo cannot be ruled out. However, unless uric acid is stable in the body, degradation of uric acid might contribute to the consumption of antioxidants. This study employed catalase to elucidate the pathway of the prooxidant effect to some extent; a more comprehensive panel including superoxide dismutase and other compounds could be developed for a more thorough investigation. Furthermore, a larger study population would allow for better segregation of patient groups with different biochemical and clinical statuses to avoid potential confounding effects.

## 5. Conclusion

Uric acid has demonstrated prooxidant properties in both in vitro and in vivo studies. Its DNA-damaging effects on lymphocytes were observed at 800 μM rather than at 600 μM. The Fenton reaction did not appear to be responsible for the DNA damage, which was likely due to hydrogen peroxide generation via an unclear mechanism. Although patients with renal failure are reported to have higher levels of DNA damage in epithelial cells, this study has shown that hyperuricemia patients with and without renal failure had similarly high levels of leukocytic DNA damage.

A correlation study suggested that serum uric acid levels were more strongly associated with DNA damage than the severity of renal failure as indicated by serum creatinine or urea, although this finding is inconclusive. The mechanism of the effect was mediated by hydrogen peroxide production, independent of the Fenton reaction and renal failure.

## Figures and Tables

**Table 1:** Demographic and laboratory data for healthy participants and those with hyperuricemia with and without renal failure. No statistically significant difference in age was observed among the three groups (Kruskal–Wallis test, p = 0.0921, followed by Dunn’s multiple comparison test, p > 0.05).

	Normal subjects (n = 20)	Hyperuricemia with renal failure subjects (n = 15)	Hyperuricemia without renal failure subjects (n = 20)
Age (years)	66.2 ± 16.7	73 ± 16.8	54.8 ± 14.8
Sex(M/F)	10M, 10F	10M, 5F	9M, 11F
Uric acid (μM)	239.8 ± 52.8	507 ± 49.5	487.1 ± 70.3
Creatinine (μM)	64.1 ± 14.6	161.7 ± 109.3	74.9 ± 9.8
Urea (mM)	5.1 ± 1.4	10.4 ± 5.4	6.0 ± 1.7

**Table 2:** Correlation matrix of comet score, age, urea, uric acid, and creatinine by multiple analysis of variance. No analyte showed a significant correlation with comet score.

	Comet Score	Age	Urea	Uric acid	Creatinine
Comet Score	1	−0.68	−0.02	0.10	−0.92
Age	−0.68	1	0.01	−0.16	0.40
Urea	−0.02	0.01	1	−0.90	−0.13
Uric acid	0.10	−0.16	−0.90	1	0.017
Creatinine	−0.92	0.40	−0.13	0.02	1
